# An Alternative Approach for Assessing Biogenicity

**DOI:** 10.1089/ast.2020.2282

**Published:** 2021-02-04

**Authors:** Joti Rouillard, Mark van Zuilen, Céline Pisapia, Juan-Manuel Garcia-Ruiz

**Affiliations:** ^1^ Laboratario de Estudios Cristalograficos, Instituto Andaluz de Ciencias de la Tierra, CSIC—Universidad de Granada, Armilla, Spain.; ^2^ Institut de Physique du Globe de Paris, Université de Paris, CNRS UMR 7154, Paris, France.

**Keywords:** Biosignatures, Biogenicity, Astrobiology, Early life, Cellular life

## Abstract

The search for signs of life in the ancient rock record, extreme terrestrial environments, and other planetary bodies requires a well-established, universal, and unambiguous test of biogenicity. This is notably true for cellular remnants of microbial life, since their relatively simple morphologies resemble various abiogenic microstructures that occur in nature. Although lists of qualitative biogenicity criteria have been devised, debates regarding the biogenicity of many ancient microfossils persist to this day. We propose here an alternative quantitative approach for assessing the biogenicity of putative microfossils. In this theoretical approach, different hypotheses—involving biology or not and depending on the geologic setting—are put forward to explain the observed objects. These hypotheses correspond to specific types of microstructures/systems. Using test samples, the morphology and/or chemistry of these systems are then characterized at the scale of populations. Morphologic parameters include, for example, circularity, aspect ratio, and solidity, while chemical parameters could include elementary ratios (*e.g.*, N/C ratio), isotopic enrichments (*e.g.*, δ13C), or chirality (*e.g.*, molar proportion of stereoisomers), among others. Statistic trends distinguishing the different systems are then searched for empirically. The trends found are translated into “decision spaces” where the different systems are quantitatively discriminated and where the potential microfossil population can be located as a single point. This approach, which is formulated here on a theoretical level, will solve several problems associated with the classical qualitative criteria of biogenicity. Most importantly, it could be applied to reveal the existence of cellular life on other planets, for which characteristics of morphology and chemical composition are difficult to predict.

## 1. Biosignatures and Biogenicity

In recent decades, a large number of scientific studies have explored the boundaries of life, be they environmental [extreme settings (Jannasch, 1985; Belilla *et al.*, 2019)], temporal [ancient rocks (Noffke *et al.*, 2006; Sugitani *et al.*, 2006)], or spatial [extraterrestrial environments (Oyama *et al.*, 1976; Des Marais *et al.*, 2008; Schwieterman *et al.*, 2018)]. In these different contexts, the search for life relies on the detection of biosignatures, that is, features characteristic of life: living organisms, the evidence of life activity in its environment, or fossils of past organisms and traces of their past activity (Des Marais *et al.*, 2008).

Defining a specific feature as a biosignature is far from straightforward. Neveu *et al.* (2018) proposed a list of instrumental and contextual criteria that must be satisfied in order for a feature to constitute a measurable convincing biosignature. These criteria are notably meant to avoid false negatives (*i.e.*, misinterpreting a biosignature as an abiotic feature or a background noise) and false positives (*i.e.*, misinterpreting an abiotic feature or a contamination as a biosignature).

According to Neveu *et al.* (2018), a good biosignature must first be generic: it should be a general feature shared by living organisms on Earth and potentially by other forms of life. Second, the environment where a biosignature is claimed to be found must be consistent with the (past) presence of life and the preservation of the biosignature. In other terms, the local habitability and preservability characteristics must be assessed (Westall *et al.*, 2015).

The hypothesis of contamination must also be discarded. When searching for extraterrestrial biosignatures, the most critical source of contamination is terrestrial organic molecules and/or organisms, brought by spatial exploration itself (Kminek *et al.*, 2014). In extreme terrestrial settings, discarding contamination means foremost proving the indigeneity of the biosignature (*i.e.*, whether the feature has originated where it is measured/sampled, *e.g.*, Santelli *et al.*, 2010). For ancient life, beyond its indigeneity (Westall and Folk, 2003), the syngenicity of the biosignature—whether the feature originated at the same time as its host rock—must also be ensured to discard contamination (*e.g.*, van Zuilen *et al.*, 2007; Rasmussen *et al.*, 2008; Javaux *et al.*, 2010).

A good biosignature must also be reliable, it must be distinguishable from similar features produced abiotically. In other words, it must be possible to assess its biogenicity.

## 2. Biogenicity: A Difficult Notion

Numerous discussions have demonstrated the difficulty of rigorously assessing biogenicity (whether a feature has been formed by life or not). We focus here on examples from early life research (Schopf, 1983; García-Ruiz, 1994; Lowe, 1994; Brasier *et al.*, 2002; van Zuilen *et al.*, 2002; McLoughlin *et al.*, 2010; Allwood *et al.*, 2018; McMahon, 2019), but similar debates exist for astrobiology (Klein, 1978; McKay *et al.*, 1996; Des Marais *et al.*, 2008; Martel *et al.*, 2012; Seager and Bains, 2015).

The reliability of most types of early life biosignatures has been debated to some extent. For example, isotopic signatures attributed to biological fractionation may also be obtained through abiotic processes (McCollom and Seewald, 2006). They do not constitute a good biosignature *per se*. Organic matter itself is not a good biosignature either, since it may be synthesized abiotically in many different environments (Chyba and Sagan, 1992; Lollar *et al.*, 2002; García-Ruiz *et al.*, 2003; Wolf and Toon, 2010; McCollom, 2013; Milesi *et al.*, 2015; Ménez *et al.*, 2018). Microbially influenced sedimentary structures (MISS), such as stromatolites, are a classical type of biosignature for early life (Hofmann *et al.*, 1999; Tice and Lowe, 2004; Allwood *et al.*, 2006; Nutman *et al.*, 2016). Yet the biogenicity of some purported Archean MISS has been repeatedly questioned and has also been inferred to represent abiotic structures deposited under specific sedimentary conditions, features of fluid escape during diagenesis, or structures resulting from shear stress and/or compression (Lowe, 1994; Awramik and Grey, 2005; McLoughlin *et al.*, 2008; Allwood *et al.*, 2018; van Zuilen, 2018).

**FIG. 1.**
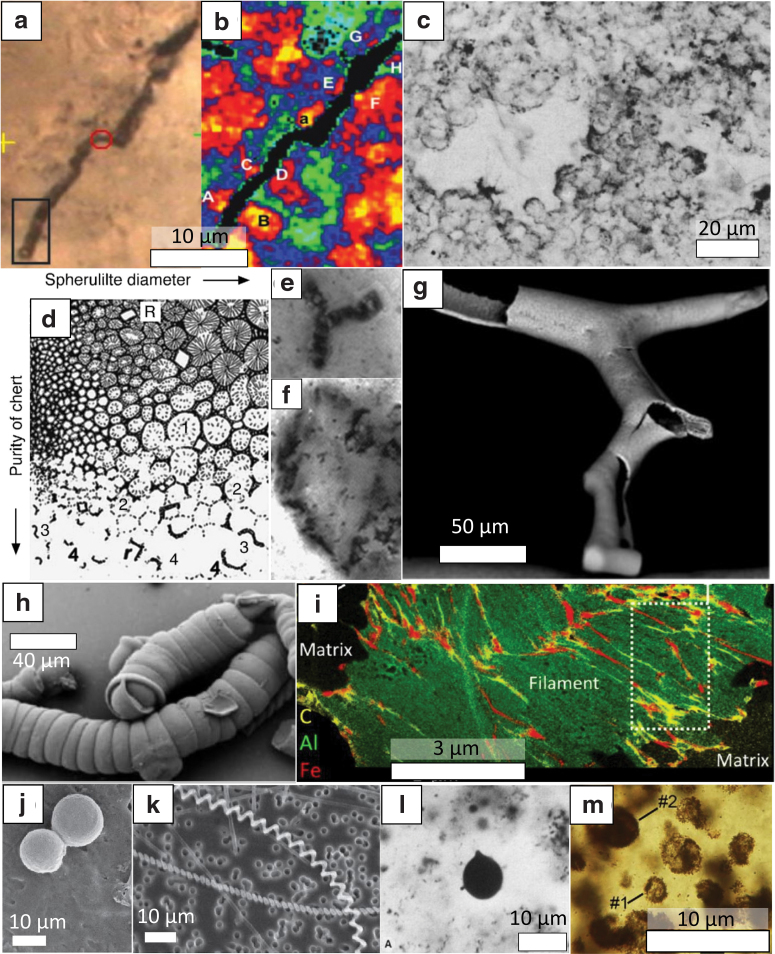
Diversity of abiogenic microfossil-like objects. **(a**, **b)** Orientation of quartz crystals around a microstructure from the 3.46 Apex chert imaged by Raman mapping and indicating that this structure was formed by cracks in the mineral lattice after recrystallization of the mineral matrix (Bower *et al.*, 2016). **(c)** Populations of siliceous spheroids clustered together and coated by organic material found in the Dresser formation (Buick, 1990). **(d**–**f)** Model **(d)** advanced to explain the formation of microstructures in the Apex chert **(e**, **f)** by the relocation of organic matter on the edges of growing silica spherulites (Brasier *et al.*, 2005). **(g)** Bifurcating siliceous tube formed experimentally by chemical gardening, that is, immersion of metal salts into a highly alkaline (pH >11) silica-rich solution (McMahon, 2019). **(h)** Filamentous silica/carbonate biomorphs synthesized experimentally by carbonate precipitation in an alkaline (pH >9) silica-rich medium (García-Ruiz *et al.*, 2003). **(i)** Stacking of phyllosilicates imaged by elementary mapping in a filamentous microstructure from the Apex chert (Wacey *et al.*, 2015). **(j**, **k)** Organomineral aggregates composed of carbon and sulfur and forming hollow spherical shells **(j)** and helical filaments **(k)** (Cosmidis and Templeton, 2016). **(l)** Organic microstructure from the Dresser formation and interpreted as a fossilized bitumen droplet (Buick, 1990). **(m)** Spheroidal hematitic aggregates from the 0.8 Chanda formation (Schopf *et al.*, 2010). Color images are available online.

Some of the most heated controversies concerning biogenicity are focused on cellular remnants of early life. The structural simplicity of early prokaryotic life-forms (*e.g.*, the absence of compartmentalization) prevents the use of comparative anatomy for phylogenetic affiliations, which is a fundamental aspect of Proterozoic and Phanerozoic paleontology. Microbial remnants in fossil assemblages have often been altered by early taphonomic events (*e.g.*, Knoll and Golubic, 1979). Besides, localities where organic microfossils have been reported are metamorphosed and often affected by hydrothermal circulation (Rasmussen, 2000; Ueno *et al.*, 2001; Brasier *et al.*, 2002; Kiyokawa *et al.*, 2006). Hydrothermal activity and metamorphism, by promoting the recrystallization of the mineral matrix and/or the circulation of organic fluids, can greatly modify the chemistry, ultrastructure, and even the general morphology of microfossils. Many abiotic objects, such as self-organized (organo-)mineral aggregates (Hopkinson *et al.*, 1998; García-Ruiz *et al.*, 2003, 2017; Cosmidis and Templeton, 2016; Muscente *et al.*, 2018; McMahon, 2019), mineral exfoliations (Wacey *et al.*, 2015), tracks of the displacement of individual mineral grains [ambient inclusion trails; (*e.g.*, Knoll and Barghoorn, 1974)], interstitial fillings (Brasier *et al.*, 2005), or bitumen droplets (Buick, 1990), are consequently difficult to distinguish from true but structurally simple and strongly altered microfossils (Fig. 1).

Several of the early reported Archean microfossils were later reinterpreted as abiotic features [see review in Schopf (1983)], and some of the more recent claims of discovery of ancient microfossils—such as those occurring in the well-studied Apex Chert, Pilbara, Western Australia, or in the Nuvvuagittuq Greenstone Belt, Quebec—are still extensively debated (Brasier *et al.*, 2002; Schopf *et al.*, 2002, 2018; García-Ruiz *et al.*, 2003; Schopf and Kudryavtsev, 2009; Marshall *et al.*, 2011; Wacey *et al.*, 2015; Dodd *et al.*, 2017; McMahon, 2019). These controversies regarding the nature of potential microfossils have particularly advanced our conceptual understanding of biogenicity.

## 3. Assessing Biogenicity Using Biogenicity Criteria—Example of Microfossil Evaluation

How can we assess the biogenicity of a specific feature found in the rock record? Is it possible to rigorously distinguish a biologic feature from its abiotic counterparts? These questions have been especially discussed for early life microfossils. In the following section, we briefly present the current protocols of biogenicity assessments for potential microfossils and illustrate them using two well-known Archean assemblages. We then present the different issues associated with these protocols.

### 3.1. Current protocols for assessing microfossil biogenicity

Since the 1980s, several articles and book chapters have presented protocols for assessing the biogenicity of potential microfossils, in which the observed microstructures must verify a list of criteria to be considered true microfossils. The null hypothesis is nonbiogenicity, until proven otherwise (here, by satisfying a list of criteria), the object is considered abiotic. The choice of criteria and their number varies greatly between the different proposed lists.

In Table 1, we present the three main lists of criteria put forward by Buick (1990), Schopf *et al.* (2010), and Brasier and Wacey (2012). By way of illustration, the three lists of criteria are applied in Table 1 to two well-documented potential microfossil assemblages: one from the 1.88 Ga Gunflint formation, whose biogenicity is widely recognized, and one from the 3.46 Ga Apex chert, whose biogenicity is strongly debated. The individual criteria from the different lists are verified in the table by using conclusions drawn in the literature (Barghoorn and Tyler, 1965; Schopf, 1993; Brasier *et al.*, 2002, 2005; Schopf *et al.*, 2002, 2018; Schopf and Kudryavtsev, 2012; Lepot *et al.*, 2017). The application of the different sets of criteria to the Gunflint and the Apex chert assemblages shows that:

**Table 1.** Summary of Different Lists of Biogenicity Criteria Proposed for the Evaluation of Microfossil Biogenicity and Verification in the Literature of the Individual Criteria in Two Proposed Microfossil Assemblages

List of criteria		Applications	
		Gunflint	Apex chert
**Buick (1990)**			
(1) Objects embedded in the rock		Yes (Barghoorn and Tyler, 1965)	Yes (Brasier *et al.*, 2002; Schopf *et al.*, 2002)
(2) Sedimentary setting		Yes (Barghoorn and Tyler, 1965)	No (Brasier *et al.*, 2002)
(3) Objects above a minimal size		Yes (Barghoorn and Tyler, 1965)	Yes (Schopf, 1993)
(4) Objects are organic		Yes (Barghoorn and Tyler, 1965)	Yes (Brasier *et al.*, 2002; Schopf *et al.*, 2002)
(5) Similar objects form a population		Yes (Barghoorn and Tyler, 1965)	Yes (Schopf, 1993)/No (Brasier *et al.*, 2005)
(6) Objects are hollow		Yes (Barghoorn and Tyler, 1965)	Yes (Schopf *et al.*, 2002)/No (Brasier *et al.*, 2002)
(7) Objects display “cellular elaboration”		Yes (Barghoorn and Tyler, 1965)	Yes (Schopf *et al.*, 2002)/No (Brasier *et al.*, 2005)
Remarks	Easy to apply in practice		
	Potential false negatives		
**Schopf *et al.* (2010)**			
(1) Species-specific, unimodal, narrow size distribution		Yes (Barghoorn and Tyler, 1965)	Yes (Schopf, 1993)/No (Brasier *et al.*, 2005)
(2) Different stages of development		Yes (Barghoorn and Tyler, 1965)	—
(3) Limited range of morphologies		Yes (Barghoorn and Tyler, 1965)	Yes (Schopf, 1993)/No (Brasier *et al.*, 2005)
(4) Distinct cell walls		Yes (Barghoorn and Tyler, 1965)	Yes (Schopf and Kudryavtsev, 2012)/No (Brasier *et al.*, 2005)
(5) Remnants of extracellular matrix		Yes (Barghoorn and Tyler, 1965)	—
(6) Filaments: sinuosity dependent on length		—	—
(7) Benthic colonies: attachment to substrate		—	—
Remarks	Precise		
	Very specific to certain types of fossils		
**Brasier and Wacey (2012)**			
(1) Geologic context viable for life		Yes (Barghoorn and Tyler, 1965)	Yes (Schopf and Kudryavtsev, 2012)/No (Brasier *et al.*, 2005)
(2) Morphology similar to modern unicellular life		Yes (Barghoorn and Tyler, 1965)	Yes (Schopf, 1993)/No (Brasier *et al.*, 2005)
(3) Evidences of biologic behavior		Yes (Barghoorn and Tyler, 1965)	Yes (Schopf, 1993)/No (Schopf, 1993)
(4) Traces of biologic metabolism		Yes (Lepot *et al.*, 2017)	Yes (Schopf *et al.*, 2018)/No (Brasier *et al.*, 2005)
Remarks	Generalizable to other contexts		
	Criteria need to be précised		

Left column: summary of different lists of biogenicity criteria proposed for the evaluation of microfossil biogenicity. Right columns: verification in the literature of the individual criteria in two proposed microfossil assemblages: one widely recognized as biogenic (from the 1.88 Ga Gunflint formation) and one whose biogenicity is very debated (from the 3.46 Ga Apex chert).

Yes: verified; No: not verified; —: not discussed.

(1) Evaluation of the biogenicity of the more recent, well-preserved Gunflint assemblage leads to the same result using the three lists of criteria—all the criteria are satisfied and biogenicity is proven.
(2) Evaluation of the biogenicity of the more ancient, hydrothermally affected Apex chert assemblage depends on the individual lists of criteria. When using the list from Buick (1990), the criterion of sedimentary setting is not satisfied—the assemblage originates from a paleohydrothermal vein—leading to a conclusion of nonbiogenicity. The two other lists do not lead to a clear conclusion on biogenicity, due to divergent views in literature. The verification of each of the biogenicity criteria from these two lists is controversial. Consequently, the scientific community still strives to reach a consensus regarding the origin of the Apex chert assemblage.

Where do these divergences in interpretation come from? How could the current protocols of biogenicity assessment be improved? These questions are discussed in the following paragraphs.

### 3.2. Explaining divergences in interpretation using the current lists of criteria

One reason for divergences in interpretation is that the biogenicity criteria in the existing lists are primarily qualitative. This may leave room for subjectivity, particularly for the criteria regarding cellular morphology or ecosystem behavior. The criterion of cellular hollowness, for example, can be interpreted in different ways, since it is not defined how thick a cell wall structure should be, relative to the overall cell dimensions. Brasier *et al.* (2002), therefore, described the Apex chert structures as solid organic aggregates, whereas Schopf and Kudryavtsev (2009) described them as clearly hollow. The need for quantification has consequently been repeatedly acknowledged in the literature. Brasier and Wacey (2012), for example, state that cellular morphology should be quantitatively distinguished from potential nonbiologic mimics. More recently, Chan *et al.* (2019) indicated the general need to build quantitative models of biologic and nonbiologic features. Neveu *et al.* (2018) also emphasized the “needs to quantify probabilities of the validity of abiotic null hypotheses.” Assessing the probability of validity for different hypotheses could be much better achieved by using quantitative criteria. In general, defining quantitative criteria would probably facilitate discussions and improve the rigorousness of biogenicity evaluation. Yet very few studies have undertaken the actual quantitative comparison of microfossils and abiotic artifacts.

Another reason for these divergences in interpretation is that many of the defined criteria can lead to biased data selection. The criterion of biologic morphology appears to be convincingly verified for some of the Apex chert structures (Schopf, 1993); however, when looking at the entire population, many shapes are inconsistent with a biologic origin (Brasier *et al.*, 2005). Choosing only a part of the data may evidently lead to interpretational biases.

### 3.3. Specific issues with the currently accepted lists of criteria

The list proposed by Buick (1990), in particular, has proven to be useful over the years for assessing the biogenicity of many Proterozoic microstructures. However, as noted by Roger Buick in his original article, for the sake of safety, his set of criteria is selective. It could therefore exclude certain microstructures that are, in effect, true microfossils (false negatives). For example, if the microfossil has been strongly morphologically altered, the criterion of cellular elaboration may not be verified. Or, if the microfossil has lost most of its constituents during its evolution and is preserved by mineral texture alone, it would be discarded by the criterion of organic nature (Westall, 1999). Another example comprises the microfossils that are found within hydrothermal chimneys (*e.g.*, remnants of chemolithotroph communities), which are excluded by the criterion of sedimentary setting. Considering that the early life fossil record is already sparse and looking for extraterrestrial life requires the investment of a great deal of resources, the potential elimination of true microfossils is problematic.

The set of criteria defined by Schopf *et al.* (2010) is precise, but also highly specific. It applies exclusively to coccoidal and filamentous microfossils. Although these morphologies represent the vast majority of reported Archean microfossil assemblages, other microfossil morphologies have also been reported, such as spindles (Sugitani *et al.*, 2007, 2009). In general, the three different tests of biogenicity contain criteria that are mainly related to modern life on Earth [*e.g.*, the criterion of cell-like morphospace as defined by Brasier and Wacey (2012)]. However, it is impossible to predict the types of morphologies that would be displayed by evolutionary distant organisms, that is, early life or extraterrestrial life.

The third list (Brasier and Wacey, 2012) has a broader purpose, since it is also designed for recognizing cellular life in extraterrestrial environments. In practice, however, the very general nature of the criteria (*e.g.*, the criterion of morphology relies on the comparison with a “cellular morphospace”) may complicate their verification as long as they are not further specified.

### 3.4. General issues with the current type of decision protocol

In a general sense, there are also issues associated with this type of decision protocol. First, it does not give equal weight to biogenic and abiogenic explanations, since the null hypothesis is nonbiogenicity. In the context of life recognition, this is meant to increase certainty in the decision. Indeed, biogenicity is considered the most “extraordinary” claim and requires “extraordinary evidence” (Carl Sagan). Underlying this is an implicit probabilistic reasoning: the hypothesis of biogenicity is believed to have a lower probability of being true than the hypothesis of nonbiogenicity. However, two major caveats exist: (1) applying probabilities should rely on a minimal contextual knowledge. For both astrobiology and early life, the environmental context is poorly understood, and therefore applying probabilistic reasoning seems quite risky. (2) The probability that should be considered is not the probability of finding a microfossil in an extraterrestrial environment, or of finding a microfossil in an Archean rock. It is the probability that the specific object considered is a true cellular remnant, which is always higher: a potential microfossil is being evaluated because it has been selected as a promising candidate. This probability is also highly variable and will take a different value for each instance. In general, it is important to devise a decision protocol where no *a priori* assumption about the likeliness of alternative hypotheses is made.

Second, this type of protocol exclusively focuses the attention of the scientific community on cellular remnants. If the microstructure does not satisfy the biogenicity criteria, no information other than its “nonmicrofossil” nature is given. However, the main question that arises when observing a potential cellular biosignature is not whether it is an actual microfossil, but what its origin—whether biologic or nonbiologic—actually is. This is particularly important in the fields of early life and astrobiology, where the amount of data is limiting; any precise information can have important implications for our understanding of these (paleo)environments. It would therefore be important to devise a decision protocol that is nonbinary and gives a more precise interpretation of the nature of objects.

Since controversies regarding the biogenicity of some microfossil assemblages persist, the current protocols of biogenicity assessment appear to be insufficient in some cases. From the different issues raised in this section, we state that an ideal protocol of biogenicity assessment should encompass the following:

(1) Be quantitative.
(2) Use criteria that apply to an entire data set—in this context, the complete population of potential microfossils—to reduce the biases associated with data selection.
(3) Be applicable in various geologic records, including strongly degraded ones, to reduce the probability of false negatives.
(4) Use criteria that are as general as possible and may also be applicable to early/extraterrestrial life.
(5) Test various precise hypotheses regarding the nature of the object, with no *a priori* assumption about their relative likelihoods.

## 4. Toward a New Protocol for Assessing Biogenicity

With the technical improvements made in recent decades, an alternative method for assessing biogenicity can be envisioned. In this section, we present how lists of classically used biogenicity criteria could be replaced by quantitative decision spaces, thus solving most of the issues discussed above.

This new method for evaluating the biogenicity of potential microfossils is introduced here using the example of morphology measurements. Subsequently, we explain how this approach can be generalized for biogenicity assessments.

### 4.1. The example of population morphometry

As was explained in the previous section, the morphology of individual microorganisms may not be considered a robust biogenicity criterion (García-Ruiz, 1999; García-Ruiz *et al.*, 2003; Cosmidis and Templeton, 2016; Muscente *et al.*, 2018; McMahon, 2019). However, at the scale of entire microbial populations, life displays a specific quantifiable morphology. For populations of abiotic objects, the morphology is only controlled by environmental (in the broader sense) constraints. For biologic populations, supplementary types of constraints exist, which are due, in large part, to (1) physiology—notably the regulation of the cell cycle that affects both individual morphology and spatial relationships among cells (Collier and Shapiro, 2007; Wang and Levin, 2009; Young, 2010; Schumacher and Søgaard-Andersen, 2017; Westfall and Levin, 2017)—and to (2) ecologic processes, which represent the ensemble of relationships (*e.g.*, exchange of matter, of information, trophic relationships, sharing of resources) that exist between individuals in a biologic population or community (White, 1836; Warming, 1895; Phillips, 1931).

Darwinian evolution implies the existence of populations of biologic individuals, and it leads therefore necessarily to ecologic processes; even the simplest forms of life on modern Earth, and probably the first cellular organisms that appeared on Earth, display rudimentary forms of ecology (Hungate, 1966; Prosser *et al.*, 2007; Egbert *et al.*, 2011; Froese *et al.*, 2014). It is a feature that is shared by, and specific to, life as we know it. When this is translated into a morphology specific to biologic populations, ecology would constitute a robust biosignature.

In one of our recent studies, we explored the potential of population morphometry for evaluating biogenicity (Rouillard *et al.*, 2019). Morphometry is the quantitative study of morphology. Using morphometry, it is possible to locate objects on morphospaces, theoretical spaces in which axes represent continuous morphology-describing parameters (Raup, 1967). Morphometry has been used previously for studying (potential) Precambrian microfossil assemblages. Size distributions are the most frequent measurements and have been used to discuss issues of paleodiversity and/or biogenicity (*e.g.*, Barghoorn and Tyler, 1965; Knoll and Golubic, 1979; Sugitani *et al.*, 2013; Köhler and Heubeck, 2019). Measurements of tangent correlation lengths (Boal and Ng, 2010) and oblateness (Sugitani *et al.*, 2018) have also led to interesting paleobiologic and paleoevolutionary interpretations.

**FIG. 2.**
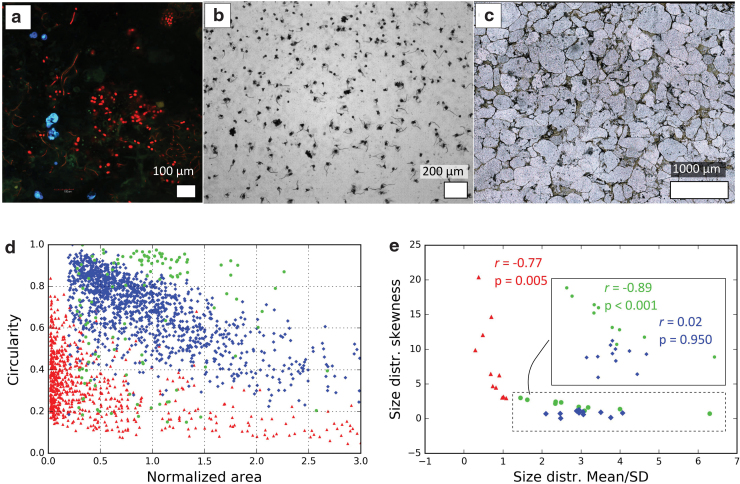
**(a**–**c)** Example populations from each of the three systems (natural microbial communities, biomorphs, and interstitial spaces in clastic rocks) compared in Rouillard *et al.* (2019). **(a)** Stromatolite-dwelling microbial community from Alchichica lake (Mexico). Picture taken using Confocal Laser Scanning Microscopy without staining. **(b)** Silica-witherite biomorphs grown in silica gel. **(c)** Interstitial spaces between quartz clasts in a sandstone filled by a dark ferruginous cement. **(d)** Relationship between area (normalized to the mean of the distribution) and circularity in the three different systems: a population of interstitial spaces (red triangles), a population of biomorphs (blue diamonds), and a microbial community (green circles). Each point represents a single particle (interstitial space, biomorph, or cellular organism). **(e)** Mean/SD of size distributions plotted against the skewness of size distribution in different populations from the same systems. The inset is a zoom-in of the area framed by the dotted line. Each point represents a population. The Pearson correlation coefficient *r* between the shapes of the different distributions and the associated probability of noncorrelation *p* are given for each system. Details about the calculation of *r* and *p* are given in the notes at the end of this article. Note the different types of correlation distinguishing the three systems. All data from Rouillard *et al.* (2019). SD, standard deviation. Color images are available online.

Rouillard *et al.* (2019) compared quantitatively the morphology of example modern communities of bacteria with the morphology of populations of two types of nonbiologic objects that could be mistaken for microfossils—interstitial spaces (*e.g.*, Brasier *et al.*, 2005) and self-assembled mineral aggregates grown in silica gel (*e.g.*, García-Ruiz *et al.*, 2003; Rouillard *et al.*, 2018). Interstitial spaces, self-assembled mineral aggregates, and microbial communities are then defined as separate systems. Each system can be represented by multiple populations (*e.g.*, the system of interstitial spaces can be represented by multiple images that each displays a different pore network in a rock). Sample images of these different systems, which correspond to different populations of objects/cells (Fig. 2a–c), were analyzed to extract parameters that describe the size and shape of individual objects and their general organization. Beyond the description of intrapopulation statistics (*e.g.*, Fig. 2d), the different populations were compared quantitatively by using nondimensional descriptors of statistic distributions (mean/standard deviation [SD], skewness, kurtosis—more details about the calculation of these parameters are presented in the notes at the end of this article).

This led to a system-level description of the different types of objects. It was found that the populations from the different systems exhibit different ranges for distribution descriptors (*e.g.*, Fig. 2e). In addition, specific correlations between population-describing parameters could be observed (Fig. 2e). Depending on the nature of items compared, distinct “levels of study” can be defined, for example, the object, the population, or multiple populations. A level of study A is higher than a level of study B if it considers individually items A1, … An, which are each composed of the items considered individually in B (*i.e.*, A1 contains B1, …, Bn). A critical result of this study is that the distinction between the systems improves with the level of study: from the level of a single object, to the level of a population of objects, and finally to the level of multiple populations.

### 4.2. From discriminant analyses to decision spaces

**FIG. 3.**
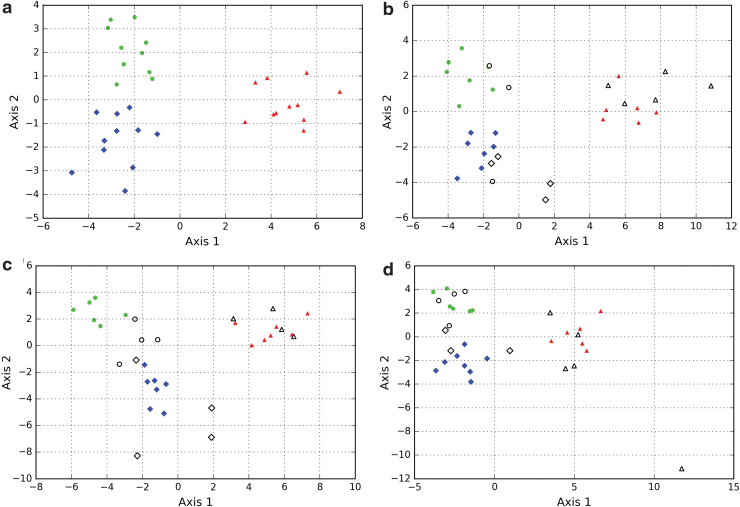
Discriminant analyses conducted on populations of interstitial spaces, biomorphs, and microorganisms. The parameters used are the different adimensional distribution descriptors (mean/SD, skewness, and kurtosis) of the statistic distributions of size, circularity, and solidity—nine parameters in total. The axes correspond to linear combinations of all parameters that allow the maximization of the variance between the different systems in a two-dimensional space (Axis-1 vs. Axis-2). **(a)** Discriminant analysis run with the complete set of populations available from the three systems. **(b**–**d)** Discriminant analyses run with only 20 populations chosen randomly out of the 32 populations available. These populations constitute a training set, shown as filled symbols. The remaining populations—the test set—are shown as hollow symbols on the obtained discriminating spaces. Note that the results of the discriminant analyses are variable depending on the training set. The discrimination achieved is imperfect, since some populations of the test sets plot away from their system of origin. This indicates that the training sets do not encompass the heterogeneity of the systems. Data used to make the analyses are from Rouillard *et al.* (2019). Color images are available online.

The morphometric characterization of populations, as described in the previous paragraph, shows that biogenicity can be evaluated by using quantitative methods. A first possible method is the use of discriminant analyses (Lachenbruch and Goldstein, 1979; McLachlan, 2004; Bishop, 2006). By applying automated discriminant analyses on the distribution descriptors from all sample populations, it was found that a good separation of the different systems (interstitial spaces, biomorphs, and microbial cells) is possible (Fig. 3a). However, an important remark must be made. To be of interest to biogenicity assessment, discrimination should be valid for any population from the different systems. However, the results of automated discriminant analyses are strongly dependent on data input (Fig. 3b–d); they find the linear combination of parameters that, at best, separate the given ensemble of populations. If the sample populations used in the study do not encompass the heterogeneity of the systems, the results cannot be used to assess biogenicity in a general context.

One way to solve this issue could be to integrate a larger amount of sample populations from more sources. This would significantly improve the description of the systems, and the applicability of the results of the discriminant analyses would be increased. However, the difficulty of accessing a large amount of data in natural contexts, and the fact that such results may not be applicable to unknown contexts (*e.g.*, extraterrestrial), means that this is not an ideal solution.

Alternatively, to obtain a more robust discrimination (less dependent on data input), we propose considering only specific parameters. The critical question concerns the choice of these parameters. These parameters should translate the specificities of life—features shared by all and only life—into a range of numeric values that distinguish the biologic system from the abiotic systems. The study of statistic morphometry hints at a promising answer. As stated earlier, it was found that the morphologic distinction between the different systems improves with the level of study (see end of Section 5.1). Single objects from the different systems cannot be reliably distinguished. At the level of populations, the different systems appear to have different types of control on shape (Fig. 2d). At the multipopulation level, the variability of statistic distributions distinguishes the different systems very consistently (Fig. 2e). We propose the hypothesis that statistic trends occurring at higher levels are due to more general processes, and that they are also more likely to be shared by all life. From this study, the degree of correlation between distribution-describing dimensions, or the slope of the linear fit of these correlations (Fig. 2e), for example, represents promising quantitative parameters for evaluating biogenicity. The origin of these differences in trends is not understood yet; it is probable that they are due to processes specific to life, such as ecology. The choice of the discriminating parameters relies here on a purely empiric analysis, making it a data-driven procedure, less prone to the human errors that could occur during data interpretation.

**FIG. 4.**
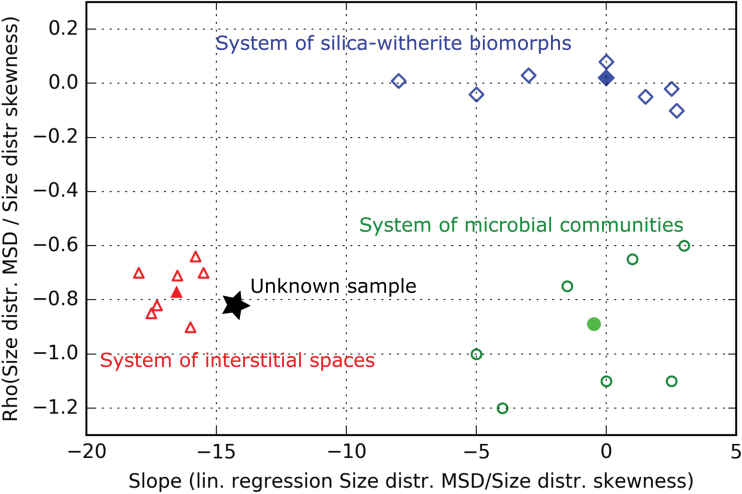
Example of a multipopulation-level decision space devised using data from Rouillard *et al.* (2019) (filled symbols; parameters plotted on Fig. 2e) and hypothetical groups of populations (hollow symbols). Values on the *y*-axis correspond to the Pearson correlation coefficient between the mean/SD and skewness of size distributions measured for the populations in the three different systems (they correspond to the *r* values given in Fig. 2e). Values on the *x*-axis represent the slopes of the linear fits between the same parameters in the three systems. The filled green circle, the filled red triangle, and the filled blue diamond therefore characterize the statistic trend found, respectively, within the group of 10 microbial communities, the group of 11 populations of interstitial spaces, and the group of 11 populations of silica-witherite biomorphs shown in Fig. 2e. Note that no statistically significant linear correlation was found between these two parameters for the 11 populations of silica-witherite biomorph populations (this could come from the overlap of distinct growth conditions in gels). A hypothetical group of populations of microstructures of unknown nature, represented by a star, may be located along these three systems in the decision space. By considering supplementary hypothetical groups of populations (hollow symbols), systems may be represented by a set of points. Numeric probabilities that the sample belongs to each of the different systems can then be given. Note that it is possible to discriminate any pair among the three systems using only one parameter, but the two parameters are required to discriminate all three systems. Color images are available online.

By using such parameters, it is therefore possible to devise decision spaces specific to the systems considered (example shown in Fig. 4, using the data shown in Fig. 2e). Decision spaces are parametric spaces in which one sample (the potential biosignature) is represented by a single point and the location of the sample enables one to make a decision regarding its nature. A “decision space,” we propose here, may rely on different types of measurements (not only measurements of morphology). For instance, some parameters may reflect trends found in individual measurements of elementary/molecular composition, of structural order, of isotopic enrichments, and so on. Decision spaces can therefore integrate an array of various information quantitatively. The number of dimensions of the space corresponds to the final number of selected parameters. In these spaces, the systems occupy defined volumes (in the wider sense) with a minimum overlap. If the data analysis has been correctly conducted, since the trends evidenced are shared at the scale of the system, such a discrimination is more robust to data input than automated discriminant analysis. Also, since it is easier to reason with single parameters than linear combinations of many parameters, the discussion of the specific parameters to be considered for the decision is easier than the discussion of the principal components obtained using discriminant analyses.

In the illustrative example of decision space shown in Fig. 4, the three systems are represented by the three groups of populations characterized in the work of Rouillard *et al.* (2019) (filled green circle, filled red triangle, and filled blue diamond). The three systems are discriminated according to the relationship between the skewness and the mean/SD of the size distributions in the system's populations (Fig. 2e), with the linear correlation coefficient between the two parameters plotted on the *y*-axis and the slope of the linear fit plotted on the *x*-axis. The three systems are correctly separated in this space. Assuming that enigmatic microstructures are discovered in a new sample, according to their geologic context, the following three hypotheses—“interstitial spaces,” “silica/carbonate biomorphs,” and “microbial community”—are put forward to explain their origin. Depending on the correlation it exhibits, the group of populations of microstructures in this sample can be located alongside these systems in the space shown in Fig. 4 (see star in Fig. 4), and it may be attributed to the closest system (interstitial spaces in this example). We note that in this specific decision space, due to the small number of populations considered in the work of Rouillard *et al.* (2019), the data available as of now for each system are plotted as one (filled) symbol, which means that it is not possible to give numeric probabilities for the sample to belong to each of the defined hypotheses/systems. By characterizing distinct groups of populations from these systems (coming, *e.g.*, from distinct natural locations/experimental settings), each system could be represented by a cloud of points (see hollow symbols in Fig. 4), thus enabling the application of statistic models to determine the probabilities of validity for each hypothesis/system.

In the following section, we expand from this example to explain how this approach can be generalized and improved for the biogenicity assessment of various biosignatures.

## 5. A General Protocol for Biogenicity Assessments Integrating Decision Spaces

5.1. When evaluating the biogenicity of a proposed biosignature, the different explanatory hypotheses should first be defined. Hypotheses depend on the geologic context, that is, what types of objects/features could be expected in such an environment? Hypotheses may involve biology or not, and to different degrees, integrating a potentially more nuanced view of biogenicity.

To illustrate this, we consider the hypothesis of the discovery of filamentous microstructures in a paleohydrothermal chimney, which is similar to structures reported in the work of Schopf (1993) and Dodd *et al.* (2017). Based on current knowledge of hydrothermal systems, a specific set of hypotheses can be made regarding the nature of these structures. They may represent (a) fossils of chemolithoautotrophs living *in situ* (Jannasch, 1985; Schuler *et al.*, 2017; Ward *et al.*, 2017); (b) dead organisms fallen from the water column and circulated by hydrothermal fluids (Duda *et al.*, 2018); (c) mineral stalks produced by the metabolism of bacteria; (d) mineral gardens grown due to chemical gradients between hydrothermal fluids and seawater (Hopkinson *et al.*, 1998; McMahon, 2019; Johannessen *et al.*, 2020); (e) biomorphic, hybrid mineral aggregates growing in hydrothermal fluids (García-Ruiz *et al.*, 2003, 2017); or (f) fillings in interstitial spaces in the mineral lattice by organic-rich fluids (Buick, 1990; Brasier *et al.*, 2005). The paleoenvironmental interpretation is important at this stage. The hypotheses formulated for structures found in a hydrothermal chimney are distinct from the hypotheses formulated for similar structures found, for example, in a sedimentary rock affected by later hydrothermal events.

We emphasize that all hypotheses have the same position in the protocol; there is no null hypothesis and no *a priori* assumption of their different respective probabilities. As such, we claim that this method follows precisely the recommendation made by Martin Brasier, that is, “that no claim for life can be accepted until all alternative abiotic explanations for its formation are discarded.” The selection of the hypotheses is critical, since reducing their number improves the quality of discrimination (fewer parameters are required to separate fewer systems, see caption of Fig. 4), but increases the risk of missing the valid hypothesis.

**FIG. 5.**
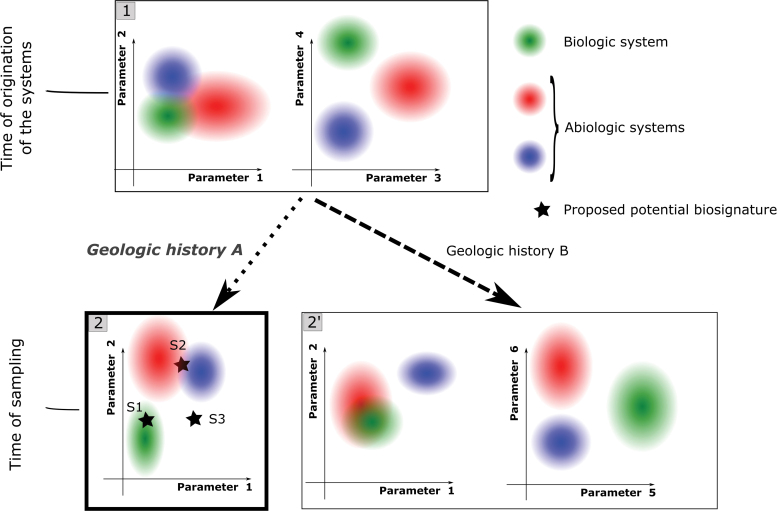
Illustration of the importance of parameter choice for assessing biogenicity using decision spaces. Here one example of a biologic system and two examples of abiotic systems are shown, located, respectively, by green, blue, and red ellipses in spaces built with couples of parameters. **(1)** Shows the systems before geologic evolution, while **(2**, **2′)** show the systems altered by geologic history (pressure, temperature, fluid circulations). The two dotted arrows indicate two different geologic histories. Depending on the history and on the parameters considered, the initial characteristics of the different systems can undergo various changes. For example, with respect to parameters 1 and 2, systems may diverge [green and blue systems from **(1)** to **(2)**] or converge [green and red systems from **(1)** to **(2′)**. Parameters chosen for biogenicity assessment are those that maximize discrimination between the systems at the end of the geologic history of the proposed biosignature. Potential biosignatures are located by stars in **(2)**. The geologic history inferred for these proposed biosignatures is A. The parameters chosen to build the decision space are consequently 1 and 2. Here, S1 may be interpreted as biogenic. S2 is interpreted as abiotic, but no information regarding its nature (blue system or red system) can be obtained. S3 is too far from any of the three systems, and no decision about its biogenicity should be made using this decision space. Statistic models may be applied to give actual probabilities of validity for the different systems. Color images are available online.

5.2. The systems (corresponding to the different hypotheses) should then be characterized exhaustively, by using test samples, along with the proposed biosignature. The goal is to characterize systems as they are at the end of the geologic history of the proposed biosignature, that is, at the time of sampling of the proposed biosignature (Fig. 5). This requires a good knowledge of local geologic history [evolution of pressure, temperature, and events of fluid circulation (Chan *et al.*, 2019), including taphonomic effects (Knoll and Golubic, 1979)] and the way this specific history would affect the candidate systems.

This knowledge may be acquired by tracing the systems in the well-known geologic record (Jones *et al.*, 2001; Guidry and Chafetz, 2003; Konhauser *et al.*, 2004; Campbell *et al.*, 2015) or by using artificial taphonomy/diagenesis/alteration experiments (Oehler, 1976; Picard *et al.*, 2015; Alleon *et al.*, 2016a, 2016b). For geologic records affected by hydrothermalism, it is crucial to know at which time of geologic history hydrothermal circulation occurred; the effects of hydrothermal fluids on nonfossilized bacteria would presumably differ from the effects of a late hydrothermal circulation on already fossilized bacteria. Although the specific example of morphologic measurements was put forward in the previous section, other types of measurements (*e.g.*, chemical maps) would improve the quality of characterization and the reliability of subsequent discrimination.

The issue of sampling is of paramount importance. To obtain statistically significant results, and also to prevent biased data selection and gerrymandering, the entire proposed biosignature should be characterized. For example, in the case of a putative biofilm with cellular remnants that is under study, all of the biofilm should be characterized. Moreover, since it is critical to search for and test statistic trends that exist at the multipopulation level, several populations of objects of a system must be measured. If the proposed biosignature occurs only in one sample, this obstacle may be overcome by spatially dividing the volume of the sample in a systematic way, thus generating several spatially segregated populations of objects. The test samples from the different systems should be characterized in a similar way, at the same scale (including spatial division of the samples) and with the same precision, so that subsequent statistical analyses can be compared.

5.3. Data obtained from the test samples are explored statistically to empirically find higher level trends (observable only at the multipopulation level) that distinguish the systems that are being studied. Specific parameters are selected that translate these distinct trends into separate numeric ranges (see parameters used in Fig. 4). Such trends may reflect classical, qualitative biogenicity criteria in a quantitative manner. For example, higher level trends found by using statistic morphometry may be linked to the criteria of “biologic morphology” and “biologic behavior.” Similar trends found through other types of quantitative measurements [*e.g.*, spatial patterns of chemicals or molecular weight distributions, see Dorn *et al.* (2011)] could reflect other classical biogenicity criteria [*e.g.*, the criteria of “biologic metabolism,” see Brasier and Wacey (2012); Kaneko and Furusawa (2018)]. The choice of the parameters depends on the geologic history (Fig. 5); the parameters chosen for the inferred geologic history (*e.g.*, Parameters 1 and 2 in Fig. 5, panel 2) may be less efficient at discriminating the different systems than other parameters if the systems were not degraded at all (compare the two graphs in panel 1) or if they underwent a different geologic history (compare the two graphs in panel 2′).

5.4. A multiparametric decision space can be built from the selected parameters, integrating potentially different types of measurements. It is clear from the preceding considerations that this decision space is not a general space that may be applied indiscriminately to any proposed biosignature. The decision space is relative to a certain type of biosignature and to a certain geologic context (Fig. 5). The nature and biogenicity (and potentially contaminant nature) of the proposed biosignature may be evaluated by simply locating its position in the decision space (stars in Fig. 5, panel 2). Such a space also allows the application of statistical models. Consequently, it is possible to calculate probabilities of validity for the different hypotheses, according to the different parameters of the decision space. It is theoretically possible that no reliable decision can be reached (*e.g.*, stars S2 and S3 in Fig. 5, panel 2). This may be due either to an insufficient discrimination between systems (star S2) or to missing the valid hypothesis (star S3).

5.5. Since decision spaces are related to a certain geologic context, beyond the nature of the proposed biosignature they can also help in assessing potential contamination issues by giving information pertaining to the syngenicity and indigeneity of the proposed biosignature. Indeed, if the feature did not originate at the same time or at the same place as the host rock, there is a high probability that it will have undergone a different geologic history; its final characteristics (assuming the characteristics of the feature are dependent on its geologic history) differ from a corresponding syngenetic and indigenous feature (compare the location of green ellipses in Fig. 5, panels 1, 2, and 2′, parameters 1 and 2). However, decision spaces should be used with caution in the context of contamination assessment, since there is a danger that a contaminant feature might overlap with one of the systems in the decision space. Contaminant systems could of course also be integrated as separated hypotheses into decision spaces, but this matter is beyond the scope of the current article.

## 6. Concluding Remarks

The protocol proposed here for assessing biogenicity represents a promising way to solve most of the issues associated with a classical protocol that relies on a list of biogenicity criteria. A number of alternative hypotheses may be defined, involving biology or not. There is no null hypothesis and no *a priori* assumption about their respective probabilities. Beyond the information pertaining to biogenicity, the use of such a protocol—depending on the specificity of hypotheses confronted—can also give precise information about the nature of the enigmatic feature that is put forward as a potential biosignature. In the context of contamination assessment, it can also help (although with a cautionary note) in evaluating the syngenicity and indigeneity of a feature. The discrimination relies on distinct trends observable only at large statistical scales, trends that may be due to general processes shared by all life. As a consequence, the possibility of biased data selection is reduced, and the protocol may be generalizable to different types of life for which absolute sizes, shapes, or chemical concentrations are unpredictable. Instead of checking qualitative, often binary criteria, this method compares the values taken by relevant biologic and abiotic systems for specific parameters; this will hopefully help the discussions and reduce subjectivity. However, the protocol proposed here requires an extensive knowledge of natural systems. Right now, decision spaces can only be built using a limited number of samples and limited types of measurements. In the future, results obtained from similar measurements on various samples from different systems should be gathered in online databases. The reliability and applicability of the protocol would thus be drastically increased.

## Notes

(1) The mean divided by SD (mean/SD), which describes the relative width of the distribution, is calculated according to the following:
  $$M∕SD\left(X\right)=\frac{\mu \left(X\right)}{\sigma \left(X\right)}$$
(2) Skewness, which describes the asymmetry of the distribution, is calculated according to the following: 
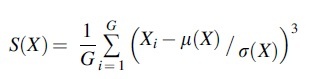
(3) Kurtosis, which describes the importance of tails in the distribution, is calculated according to the following: 
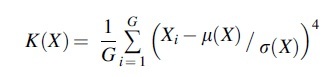
The linear correlation coefficient *r* between two parameters *X* and *Y*, measured on a population of *G* points, is calculated according to the following:
  $$r\left(X,Y\right)=\frac{\sum_{i=1}^{G}\left(X_{i}-\mu \left(X\right)\right)\left(Y_{i}-\mu \left(Y\right)\right)}{\sigma \left(X\right)\sigma \left(Y\right)}$$
The significance of the correlation *p* (or probability that the two parameters are not correlated) varies with the number of points *G* and is calculated with a two-tailed t test according to the following:
  $$p\left(X,Y\right)=r\left(X,Y\right)\sqrt{\frac{G-2}{1-r{\left(X,Y\right)}^{2}}}$$

No issue related to ethics exist for the research presented in this article.

## Abbreviations Used

MISS: microbially influenced sedimentary structures
SD: standard deviation

## Notes

Associate Editor: Michael C. Storrie-Lombardi
